# 5-ALA/SFC Ameliorates Endotoxin-Induced Ocular Inflammation in Rats by Inhibiting the NF-κB Signaling Pathway and Activating the HO-1/Nrf2 Signaling Pathway

**DOI:** 10.3390/ijms24108653

**Published:** 2023-05-12

**Authors:** Yuya Otaka, Kazutaka Kanai, Arisa Mori, Daiki Okada, Noriaki Nagai, Yohei Yamashita, Yoichiro Ichikawa, Kazuki Tajima

**Affiliations:** 1 Department of Small Animal Internal Medicine II, School of Veterinary Medicine, Kitasato University, 35-1 Higashi-23ban-cho, Towada 034-8628, Aomori, Japan; dv20001@st.kitasato-u.ac.jp (Y.O.); tajima.kazuki@kitasato-u.ac.jp (K.T.); 2 Faculty of Pharmacy, Kindai University, 3-4-1 Kowakae, Higashiosaka 577-8502, Osaka, Japan; nagai_n@phar.kindai.ac.jp

**Keywords:** HO-1, lipopolysaccharide, Nrf2, NF-κB, sodium ferrous citrate, uveitis, 5-aminolevulinic acid

## Abstract

Sodium ferrous citrate (SFC) is involved in the metabolism of 5-aminolevulinic acid (5-ALA) and enhances its anti-inflammatory effects. The effects of 5-ALA/SFC on inflammation in rats with endotoxin-induced uveitis (EIU) have yet to be elucidated. In this study, during lipopolysaccharide injection, 5-ALA/SFC (10 mg/kg 5-ALA plus 15.7 mg/kg SFC) or 5-ALA (10 or 100 mg/kg) was administered via gastric gavage, wherein we saw that 5-ALA/SFC ameliorated ocular inflammation in EIU rats by suppressing clinical scores; by infiltrating cell counts, aqueous humor protein, and inflammatory cytokine levels; and by improving histopathological scores to the same extent as 100 mg/kg 5-ALA. Immunohistochemistry showed that 5-ALA/SFC suppressed iNOS and COX-2 expression, NF-κB activation, IκB-α degradation, and p-IKKα/β expression, and activated HO-1 and Nrf2 expression. Therefore, this study has investigated how 5-ALA/SFC reduces inflammation and revealed the pathways involved in EIU rats. 5-ALA/SFC is shown to inhibit ocular inflammation in EIU rats by inhibiting NF-κB and activating the HO-1/Nrf2 pathways.

## 1. Introduction

Subcutaneous injection of lipopolysaccharide (LPS) leads to endotoxin-induced uveitis (EIU) in rats, an animal model of acute anterior uveitis [1]. In this model, inflammatory cells infiltrate the disrupted blood-aqueous barrier, and proteins leak into the aqueous humor (AqH). Acute inflammatory responses start 4 h after LPS injection and peak in 18–24 h [2,3]. EIU is a commonly used animal model for studying drug pharmacological and immunological effects on intraocular inflammation [4,5,6]. It has been suggested that the elevated expression of inflammatory cytokines, such as TNF-α, IL-1β, IL-6, NO produced by iNOS, and PGE2 produced by COX-2, are significant in the pathogenesis of EIU [7,8,9]. Furthermore, inflammatory cells infiltrating the iris ciliary body (ICB) are known to be involved in the production of these cytokines [10,11]. The expression of these cytokines has been shown to be regulated by NF-κB in intraocular tissues, including ICB [12,13,14,15].

NF-κB, a dimer of p50, and p65 maintain a quiescent state in the cytoplasm by binding to IκB-α to prevent nuclear translocation signals [16,17]. Upon stimulation with LPS, IκB-α is phosphorylated by p-IKK and degraded, and the p65 subunit is transferred to the nucleus, where it induces inflammatory gene expression [18]. IKK consists of three subunits (IKKα, IKKβ, and IKKγ) and IKKβ is mainly involved in IκB-α phosphorylation. Inhibiting NF-κB activation—p65 nuclear translocation—may be a therapeutic target for ocular inflammation, where NF-κB activation is considered essential to EIU pathogenesis because NF-κB inhibitors reduce inflammation in EIU [19,20,21,22].

HO-1 is a hematolytic enzyme induced by several stimuli, including LPS, oxidative stress, and ischemia-reperfusion [23,24]. Ohta et al. reported that hemin increases HO-1 expression in ICB and suppresses ocular inflammation in the EIU rats [25]; thus, the heme degradation products from HO-1, biliverdin, and iron, producing carbon monoxide, also contribute to anti-inflammation [26,27]. The anti-inflammatory effects of HO-1, which prevent nuclear translocation of NF-κB and suppress the production of inflammatory mediators [28], are thought to be due to the suppression of IκB-α degradation. Thus, enhancing HO-1 expression is considered anti-inflammatory. HO-1 is mainly expressed via Nrf2 [29], which remains quiescent in the cytoplasm and translocates into the nucleus when stimulated by LPS or other agents to activate antioxidant-responsive gene sequences such as HO-1 [30].

5-Aminolevulinic acid (5-ALA) has been detected in foods and has attracted attention in recent years due to its diverse actions [31,32,33]. In animal cells, 5-ALA synthesis is mediated by the mitochondrial aminolevulinic acid synthase from glycine and succinate CoA. Porphyrinogens, precursors of protoporphyrin IX (PpIX), are formed by condensation polymerization of eight molecules of 5-ALA. Heme is formed in the mitochondria of PpIX by inserting iron ions [34]. Interestingly, a combination of sodium ferrous citrate (SFC) and 5-ALA (5-ALA/SFC) has been suggested to be effective in rats, mice, and humans [35,36,37,38,39]. Recently, although the effects of 5-ALA/SFC have not been elucidated in this model, we found that 5-ALA alone exhibits potent anti-inflammatory effects by suppressing anti-inflammatory mediators in EIU rats [40].

Previous studies have demonstrated the HO-1 expression upregulation function of 5-ALA/SFC compared to 5-ALA [31,41]. Additionally, 5-ALA/SFC has been shown to suppress NF-κB activation and subsequent inflammatory mediator expression in a murine fatty liver ischemia-reperfusion model [42]. With this study aiming to investigate the anti-inflammatory effects of 5-ALA/SFC and elucidate the pathways involved in EIU rats, which are yet to be elucidated, we hypothesize that the 5-ALA/SFC mechanism may also be related to the NF-κB and HO-/Nrf2 pathways in EIU rats.

## 2. Results

### 2.1. 5-ALA/SFC Suppresses Clinical Scoring and the Number of Infiltrating Cells and Protein Concentration in AqH

**Figure 1:**
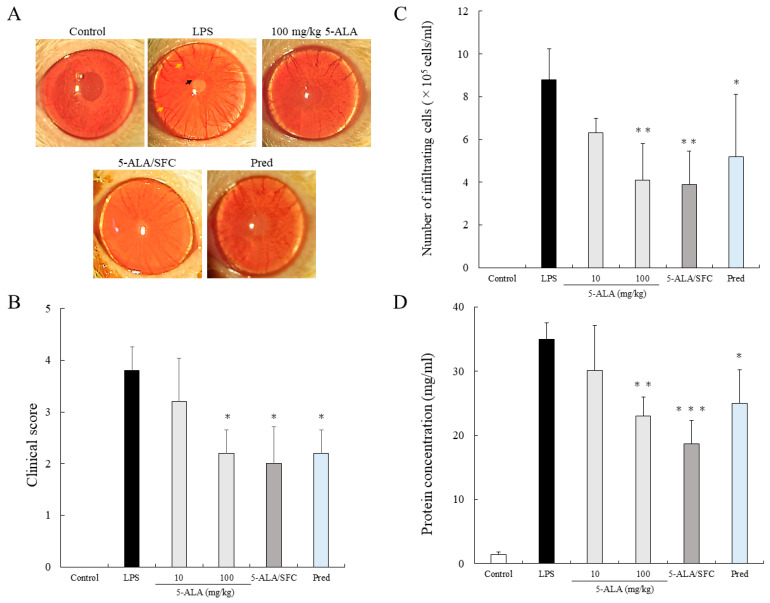
5-ALA/SFC suppressed anterior segment inflammation in EIU rats. (**A**) Representative image of the eye (black arrow: fibrinous membrane formation, yellow arrow: iridal hyperemia). (**B**–**D**) Effects of 5-ALA/SFC on a clinical score (**B**), infiltrating cells (**C**), and protein concentration (**D**) in AqH 24 h after LPS injection. Each value represents the mean ± SD (n = 5). * *p* < 0.05, ** *p* < 0.01, *** *p* < 0.001, compared with the LPS group.

No inflammatory reaction was observed in the control group. In the LPS group, the clinical score was 3.8 ± 0.45 (mean ± SD, n = 5), and intense iridal hyperemia and fibrous exudates were observed in the pupillary area. The clinical scores were 3.2 ± 0.84, 2.2 ± 0.45 (*p* < 0.05, vs. LPS), 2.0 ± 0.71 (*p* < 0.05, vs. LPS), and 2.2 ± 0.45 (*p* < 0.05, vs. LPS) when treated with 10 or 100 mg/kg of 5-ALA, 5-ALA/SFC (10 mg/kg 5-ALA plus 15.7 mg/kg SFC), and 1 mg/kg Prednisolone (Pred), respectively. The 5-ALA/SFC, 100 mg/kg 5-ALA, and 1 mg/kg Pred alleviated iridal hyperemia, and no fibrous exudates were observed. No differences were found between the clinical scores of 100 mg/kg 5-ALA, 5-ALA/SFC, and Pred (Figure 1A,B).

The number of inflammatory cells in the LPS groups was 8.8 ± 1.44 × 10^5^ cells/mL (n = 5). Treatment with 100 mg/kg 5-ALA, 5-ALA/SFC, and Pred showed a reduction in the number of infiltrating cells (100 mg/kg 5-ALA; 4.1 ± 1.71 × 10^5^ cells/mL, *p* < 0.01, 5-ALA/SFC; 3.9 ± 1.57 × 10^5^ cells/mL, *p* < 0.01, Pred; 5.2 ± 2.92 × 10^5^ cells/mL, *p* < 0.05, vs. LPS). The 5-ALA/SFC had a similar effect on AqH cell infiltration as that of 100 mg/kg 5-ALA and Pred (Figure 1C).

The AqH protein concentration in the LPS group was 34.93± 2.54 mg/mL (n = 5). Treatment with 100 mg/kg 5-ALA, 5-ALA/SFC, and Pred significantly suppressed the increase in the protein concentrations in AqH (100 mg/kg 5-ALA; 22.98 ± 2.98 mg/mL, *p* < 0.01, 5-ALA/SFC; 18.71 ± 3.65 mg/mL, *p* < 0.001, Pred; 25.04 ± 5.21 mg/mL, *p* < 0.05). Pred and 100 mg/kg 5-ALA had similar effects on AqH protein concentrations. Treatment with 10 mg/kg 5-ALA showed a slight reduction in protein concentration (30.07 ± 7.09 mg/mL) and did not differ significantly from the LPS group (Figure 1D).

### 2.2. 5-ALA/SFC Improves Histopathologic Evaluation

**Figure 2:**
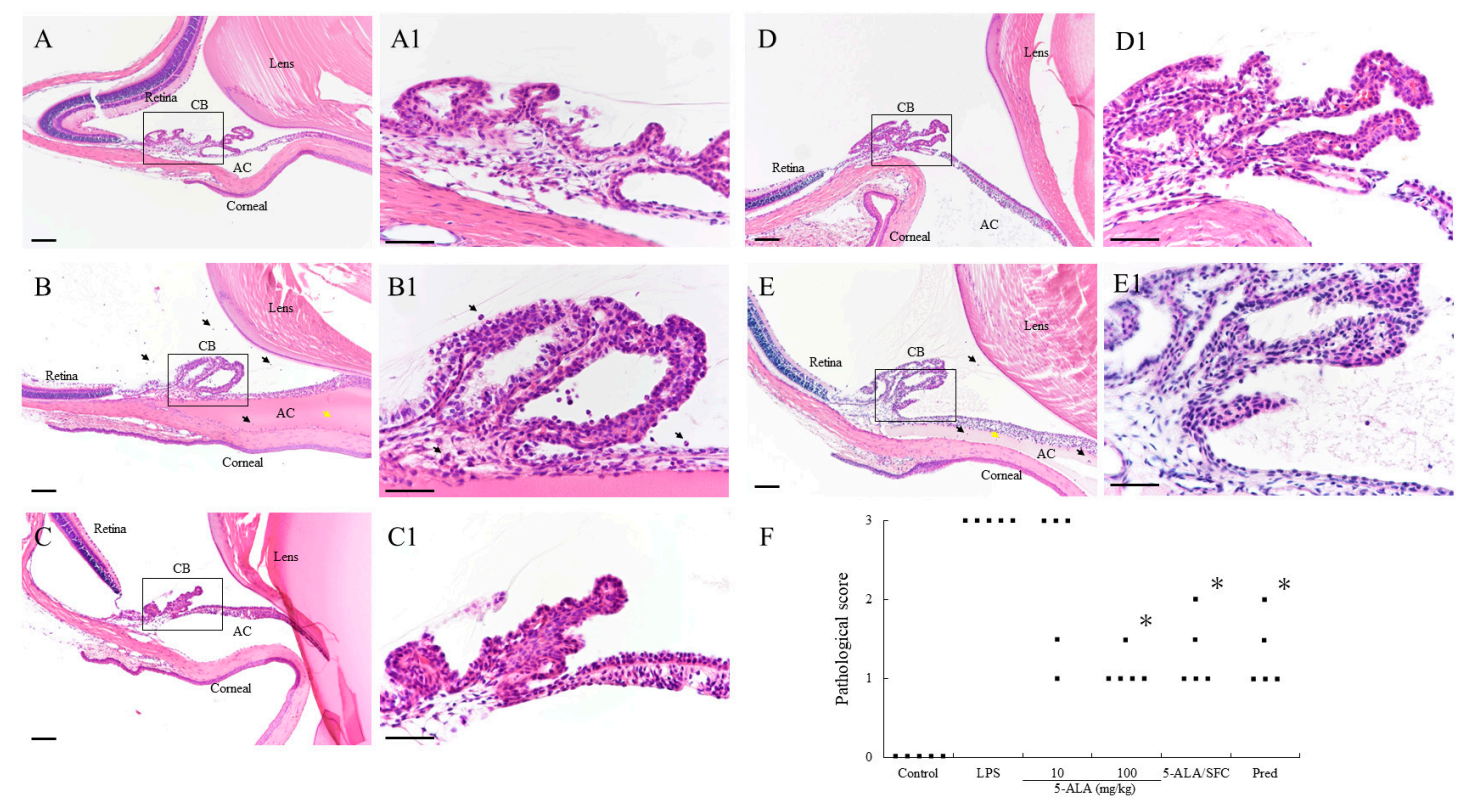
Histological evaluation of EIU rats treated with 5-ALA/SFC in the anterior segment of the eye in rats 24 h after LPS injection. (**A**,**A1**) There was no sign of infiltration in the control group. (**B**,**B1**) In the LPS group, there was a severe inflammatory cellular infiltrate in the iris stroma, CB, and AC, and a large volume of exudate with dense protein aggregation in the AC. (**C**–**E**) In the anterior segment of rats treated with 100 mg/kg 5-ALA, 5-ALA/SFC, and the Pred group, inflammatory cells, and protein exudates were reduced. (**A1**–**E1**) These are enrarged version of the area in black square in (**A**–**E**). Black arrows mark infiltrating cells. Yellow arrows mark dense protein exudation. Hematoxylin and eosin staining, H&E staining. Original magnification: (**A**–**E**) ×100, Bars, 100 µm; (**A1**–**E1**) ×400, Bars, 50 µm; AC, anterior chamber; CB, ciliary body. (**F**) Effect of 5-ALA/SFC on the histologic grading of EIU. Points indicate each rat’s means of histopathologic grading (n = 5). * *p* < 0.05, compared with the LPS group. EIU, endotoxin-induced uveitis. Pred, prednisolone.

The LPS group showed severe uveitis 24 h after LPS injection, with a 3.0 ± 0.0 (n = 5) mean histopathological grading. Treatment with 100 mg/kg 5-ALA, 5-ALA/SFC, and Pred resulted in significantly decreased histopathological scores (100 mg/kg 5-ALA; 1.1 ± 0.22, *p* < 0.05, 5-ALA/SFC; 1.3 ± 0.45, *p* < 0.05, Pred; 1.3 ± 0.45, *p* < 0.05, vs. LPS). The mean histopathologic gradings were milder after treatment with 10 mg/kg 5-ALA than in the LPS group (10 mg/kg 5-ALA: 2.3 ± 0.97), and there were no significant differences (Figure 2).

### 2.3. 5-ALA/SFC Suppresses TNF-α, IL-6, NO, and PGE2 Levels in AqH

**Figure 3:**
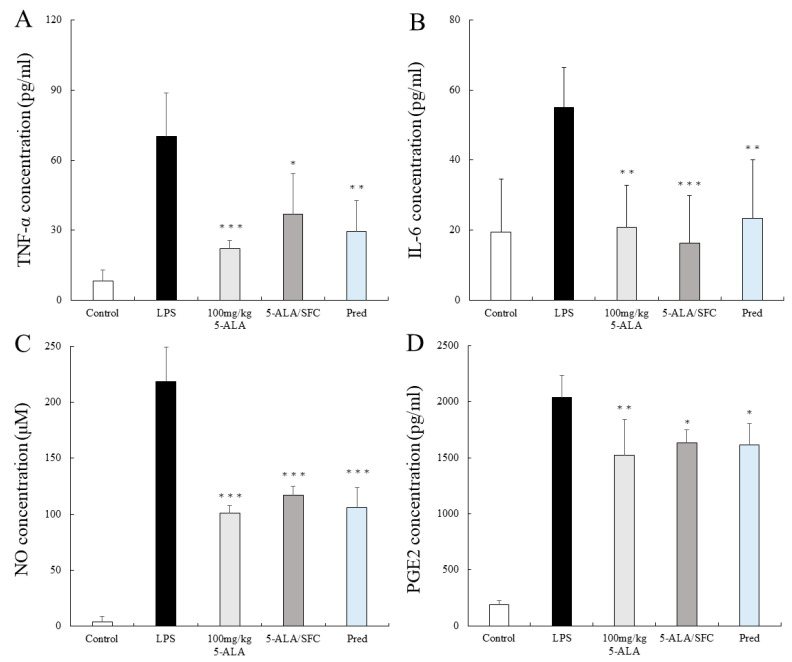
5-ALA/SFC suppressed (**A**) TNF-α, (**B**) IL-6, (**C**) NO, and (**D**) PGE2 levels in the AqH at 24 h after LPS injection. Each value represents the mean ± SD (n = 5). * *p* < 0.05, ** *p* < 0.01, and *** *p* < 0.001, compared with the LPS group.

In the LPS group, the TNF-α, IL-6, NO, and PGE2 levels were remarkable as follows: 70.11 ± 2.46 pg/mL, 54.95 ± 11.41 pg/mL, 218.71 ± 30.48 μM, and 2038.18 ± 197.32 pg/mL (n = 5), respectively. TNF-α, IL-6, NO, and PGE2 levels were suppressed by 100 mg/kg 5-ALA and 5-ALA/SFC and were not significantly different from the effect of Pred (100 mg/kg 5-ALA; 21.99 ± 3.73 pg/mL, *p* < 0.001, 20.75 ± 12.04 pg/mL, *p* < 0.01, 101.05 ± 7.01 μM, *p* < 0.001, and 1520.82 ± 322.20 pg/mL, *p* < 0.01, 5-ALA/SFC; 36.96 ± 17.34 pg/mL, *p* < 0.05, 16.29 ± 13.59 pg/mL, *p* < 0.001, 116.78 ± 8.51 μM, *p* < 0.001 and 1632.77 ± 115.17 pg/mL, *p* < 0.05, vs. LPS). Pred lowered the TNF-α, IL-6, NO, and PGE2 levels significantly (29.57 ± 13.20 pg/mL, *p* < 0.01, 23.40 ± 16.72 pg/mL, *p* < 0.01, 105.69 ± 18.04 μM, *p* < 0.001 and 1613.33 ± 192.25 pg/mL, *p* < 0.05, vs. LPS) (Figure 3A–D).

### 2.4. 5-ALA/SFC Downregulates the Expression of iNOS and COX-2 in ICB

**Figure 4:**
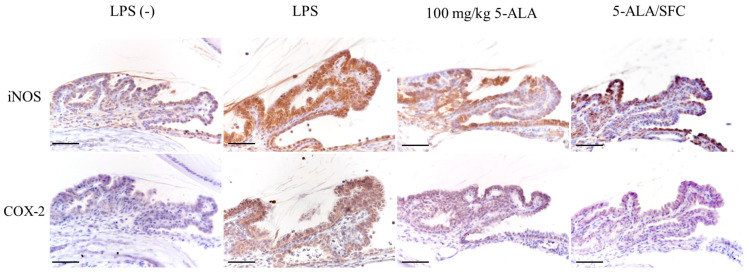
5-ALA/SFC prevented the expression of iNOS and COX-2 in the ICB 24 h after LPS injection. 5-ALA/SFC and 100 mg/kg 5-ALA suppressed iNOS and COX-2 expression compared to the LPS group. Bars: 100 µm.

The iNOS and COX-2 expression in the ICB of the LPS group increased 24 h after the LPS injection compared to the control group. Inhibition of iNOS and COX-2 expression by 5-ALA (100 mg/kg 5-ALA) and 5-ALA/SFC (100 mg/kg) was observed in ICB. Minimal iNOS and COX-2 expression was observed in the control group (Figure 4).

### 2.5. 5-ALA/SFC Downregulates the NF-κB Pathway

**Figure 5:**
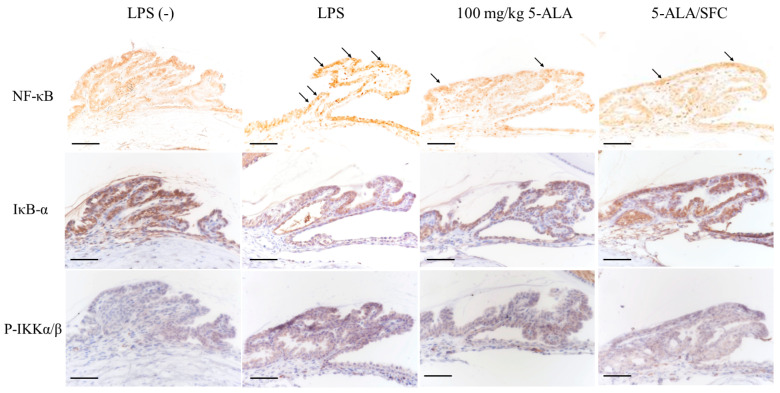
5-ALA/SFC regulates the NF-κB pathway at 3 h after LPS injection. 5-ALA/SFC inhibited the degradation of IκB-α and p-IKKα/β and suppressed the expression of activated NF-κB p65 positive cells in ICB. Bars: 100 µm. Arrows: activated NF-κB p65 positive cells.

Immunoreactivity of activated NF-κB p65 was strongly expressed, and nuclear translocation was observed in the LPS group ICB 3 h after LPS injection. 5-ALA/SFC inhibited NF-κB p65 expression in ICB. No activated NF-κB p65-positive nuclei were detected in the control group ICB. IκB-α degradation and p-IKKα/β expression in ICB were inhibited by 5-ALA/SFC. In 100 mg/kg doses, 5-ALA regulated the NF-κB pathway similarly to 5-ALA/SFC (Figure 5).

### 2.6. 5-ALA/SFC Upregulates the Nrf2/HO-1 Pathway

**Figure 6:**
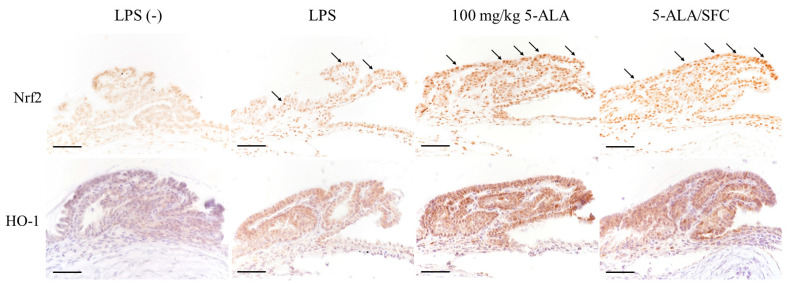
5-ALA/SFC activated the HO-1/Nrf2 pathway in the ICB 3 h after LPS injection. 5-ALA/SFC activated Nrf2 and increased HO-1 expression in ICB more than in the LPS group. Bars: 100 µm. Arrows: activated Nrf2 positive cells.

Immunoreactivity of activated Nrf2 and HO-1 was strongly expressed, and nuclear translocation was observed in the LPS group ICB 3 h after LPS injection. 5-ALA/SFC tended to activate Nrf2 and HO-1 expression in ICB compared to the LPS group. Minimal expression of Nrf2 and HO-1 in ICB was observed in the control group. With 100 mg/kg doses, 5-ALA regulated the Nrf2/HO-1 pathway similarly to 5-ALA/SFC (Figure 6).

## 3. Discussion

This study demonstrates that 5-ALA/SFC is as effective as 100 mg/kg 5-ALA in reducing inflammation in EIU. The 5-ALA/SFC suppressed the clinical scores, number of infiltrating cells, and protein leakage into the AqH. In addition, it inhibited inflammatory cytokines (IL-6 and TNF-α) and mediators (PGE2 and NO) released into the AqH, and, in a manner comparable to 100 mg/kg 5-ALA alone and 1 mg/kg of prednisolone, improved histopathologic scores. 5-ALA/SFC inhibited NF-κB p65 nuclear translocation, IKKα/β phosphorylation, and IκB-α degradation in the ICB of EIU rats. In addition, in results suggesting that 5-ALA/SFC inhibits EIU development by NF-κB signaling suppression and Nrf2/HO-1 signaling activation, 5-ALA/SFC promoted nuclear translocation of Nrf2 in the ICB of EIU rats, which increased HO-1 expression. A similar mechanism was observed with 100 mg/kg 5-ALA.

The features of EIU include blood–ocular barrier disruption and increased total protein levels in the anterior chamber [4]. Thus, total protein concentration, infiltrating cells, and inflammatory cytokines in AqH indicate the severity of inflammation. Inflammatory cytokines, released by a wide variety of inflammatory cells, such as TNF-α, IL-6, NO, and PGE2, participate in EIU pathogenesis [43]. Our previous study revealed that 100 mg/kg 5-ALA has anti-inflammatory effects on EIU rats by inhibiting the subsequent production of inflammatory mediators [40], while this study demonstrated that 5-ALA/SFC could significantly attenuate TNF-α, IL-6, NO, and PGE2 production in the AqH, thereby indicating that 5-ALA/SFC suppressed these cytokines to the same extent as 100 mg/kg 5-ALA. Furthermore, this suggests that 5-ALA/SFC is more synergistic than the additive effect of 5-ALA alone.

NO and PGE2 play a role in EIU pathogenesis [10,44]. In rats, LPS injection increases iNOS and COX-2 expression in the ICB and is involved in NO and PGE2 production [2,45]. Inhibition of NO and PGE2 synthesis may have a therapeutic effect on uveitis due to the additive effects of iNOS/NO and COX-2/PGE2 synthesis in EIU [46]. 5-ALA/SFC suppressed iNOS and COX-2 expression in the ICB of EIU rats, and the levels of NO and PGE2 in AqH might be attributed to iNOS and COX-2 suppression in the ICB.

NF-κB activity is increased in the ICB of EIU rats, leading to the development of EIU due to the excessive production of inflammatory mediators in the ocular tissues [47,48]. Several reports show that NF-κB inhibitors prevent uveitis in EIU rats [49,50,51]. The 5-ALA may reduce NF-κB activity in a rat model of ischemia-reperfusion injury [41]. In this study, our results indicate that, by suppressing NF-κB activation, 5-ALA/SFC reduced iNOS and COX-2 protein expression and, subsequently, NO, and PGE2 production, with us having showed, for the first time, that 5-ALA/SFC suppresses LPS-induced phosphorylation of the IKK, degradation of IκB-α, and the subunit of NF-κB, thereby preventing further NF-κB translocation in ICB.

Enhanced HO-1 expression early after LPS injection is considered an important therapeutic target for uveitis [25], with HO-1 exhibiting anti-inflammatory effects in the EIU rat model. In this study, 5-ALA/SFC enhanced HO-1 expression in ICB 3 h after LPS injection in the EIU rat model, further suggesting that part of the anti-inflammatory mechanism of 5-ALA/SFC in the EIU rat model is its ability to enhance the nuclear translocation of Nrf2 and consequent HO-1 expression. Thus, 5-ALA/SFC enhanced the nuclear translocation of Nrf2 3 h after LPS injection. In addition, HO-1 has been reported to inhibit the degradation of IκB-α [26]. In the present study, 5-ALA/SFC enhanced HO-1 expression and inhibited IκB-α degradation in ICB 3 h after LPS injection, suggesting that the subsequent inhibition of NF-κB nuclear translocation may be involved in early HO-1 expression.

In this study, EIU was suppressed by administering 5-ALA/SFC simultaneously with EIU induction. To clarify the therapeutic effect of 5-ALA/SFC on ocular inflammation, additional experiments are needed, such as administering 5-ALA/SFC after inducing EIU. Therefore, further experiments with 5-ALA/SFC in EIU 4 h after LPS injection are needed.

In conclusion, this study suggests that 5-ALA/SFC treatment on EIU attenuates ocular inflammation by inhibiting the expression and release of inflammatory mediators and inhibiting the activated NF-κB and Nrf2/HO-1 pathways in ICB. Therefore, it is seen that 5-ALA/SFC has been demonstrated to be effective in uveitis, and further research may yield a novel treatment for uveitis.

## 4. Materials and Methods

### 4.1. Animals

The study used 180 male Sprague Dawley rats (6-week-old, 180–220 g). The rats were purchased from SLC (Hamamatsu, Japan) and maintained in an air-conditioned room with a 12 h light/12 h dark cycle. The ARVO Statement on the Care and Use of Laboratory Animals guided the experiments in this study. This study was approved by the Animal Care and Use Committee of Kitasato University (No. 20-068), and sample size calculations were carried out using the open-source software R version 4.2.2 [52] before the study began, confirming that the study had sufficient statistical power to detect between-group differences.

### 4.2. Antibodies and Reagents

Antibodies against iNOS (ab3523), Cox-2 (ab179800), HO-1 (ab13243), and IκB-α (ab32518) were purchased from Abcam (Cambridge, UK). NF-κB p65 (sc-372) was purchased from Santa Cruz Biotechnology (Santa Cruz, CA, USA). Phosphorylated (p)-IKKα/β (#2697) was purchased from Cell Signaling Technology (Danvers, MA, USA). Nrf2 (bs-1074R) was purchased from Bioss Antibodies (Woburn, MA, USA). 5-ALA was provided by Neopharma Japan (Tokyo, Japan). SFC was obtained from Komatsuya Corporation (Osaka, Japan). LPS from *Salmonella typhimurium* and Pred were purchased from Sigma-Aldrich (St. Louis, MO, USA).

### 4.3. Induction of EIU Rats

EIU was injected subcutaneously into each footpad with 200 µg LPS diluted in 0.2 mL saline sterilized (100 µg each subcutaneously) under anesthesia with isoflurane (Mylan Inc., Canonsburg, PA, USA). In the 10 m/kg 5-ALA, 100 mg/kg 5-ALA, 5-ALA/SFC groups (10 mg/kg 5-ALA plus 15.7 mg/kg SFC), and the 1 mg/kg Pred group, each reagent dissolved in 10 mL/kg saline were administered intragastrically to each rat at the time of LPS injection. With the dose ratio of 5-ALA/SFC determined with reference to a previous study [35], in the LPS group, each rat received 10 mL/kg saline in the same schedule. In the control group, each rat received the same volume of saline and was injected subcutaneously with 0.2 mL saline without LPS. All rats were randomly divided into groups comprising five animals each.

### 4.4. Clinical Scoring

The clinical manifestations were recorded in images using a digital camera (^®^PENTAX Q-S1, RICOH Imagining, Co., Ltd., Tokyo, Japan) and evaluated in the left eye by two blinded observers 24 h after the LPS injection and before euthanization. The clinical scores were evaluated from 0 to 4 according to a previous study [53].

### 4.5. Number of Infiltrating Cells and Proteins in AqH

This analysis was performed as described in our previous report [39]. In brief, rats were euthanized with an overdose of isoflurane 24 h after LPS injection. AqH was collected from both eyes using a 30-gauge needle under the surgical microscope (OME-1000; Olympus Optical Co., Ltd., Tokyo, Japan). AqH samples from the control group were not diluted, whereas those from other groups were diluted 10-fold with sterile saline. AqH samples were added in equal parts to the Türk stain solution and counted using a hematocytometer (Bürker-Türk hemocytometer; Erma Inc., Tokyo, Japan). After counting, AqH samples were centrifuged (2500 rpm, 5 min, 4 °C). The supernatants were diluted 5 fold and 100 fold with sterilized saline in the control and other groups, respectively. A bicinchoninic acid (BCA) protein assay kit (Pierce, Rockford, IL, USA) was used to measure the total protein concentration in AqH.

### 4.6. Histopathologic Evaluation

Rats were euthanized 24 h after the LPS injection. Both eyes were enucleated under a surgical microscope, fixed in 4% paraformaldehyde in PBS for 24 h at 4 °C, and embedded in paraffin. Sagittal sections (3 µm) were cut near the optic nerve head and stained with hematoxylin and eosin (H&E). Light microscopy was used to observe ICB masked in the anterior chamber. The histopathologic evaluation of inflammation was graded 0–3, as described previously [35,40]. Both eyes of the five rats from each group were analyzed.

### 4.7. The Levels of TNF-α, IL-6, PGE2, and NO in AqH

The TNF-α, IL-6, and PGE2 levels in the AqH 24 h after LPS injection were assessed using a commercially available ELIS kit (TNF-α: KE20001; Proteintech Group Inc., Rosemont, IL, USA; IL-6: #BMS625; Thermo Fisher Scientific, Waltham, MA, USA; PGE2: 500141; Cayman Chemical Co., Ann Arbor, MI, USA) according to the manufacturer’s instructions (n = 5). The total nitrate/nitrite in AqH was measured using a NO_2_/NO_3_ colorimetric assay kit (NK05; Dojindo Molecular Technologies Inc., Kumamoto, Japan) (n = 5).

### 4.8. Immunohistochemical Studies

At 3 or 24 h after LPS injection, both eyes were enucleated under the surgical microscope, fixed in 4% paraformaldehyde in PBS for 24 h at 4 °C, and embedded in paraffin. Sagittal sections (3 µm) were obtained proximal to the optic nerve. Serial sections embedded in paraffin were deparaffinized in xylene, rehydrated using a graded ethanol series, and washed with Tris-buffered saline (TBS, pH 7.6). Antigen activation treatment was performed on each antibody using the following method. Sections using anti-iNOS antibody and anti-NF-κB p65 antibody were immersed in sodium citrate buffer (pH 6.0) and heat-treated in a microwave oven for 10 min, while sections with anti-Nrf2 and anti-IκB-α antibodies were immersed in sodium citrate buffer (pH 6.0) and heat-treated in an autoclave at 121 °C for 10 min. Sections using anti-COX-2, anti-HO-1, and anti-p-IKKα/β antibodies were immersed in antigen activator buffer (pH 9.0, Agilent, Santa Clara, CA, USA) and heat-treated in an autoclave at 121 °C for 10 min. Treatment with 3% hydrogen peroxide for 20 min blocked endogenous peroxidase activity. Blocking with 10% goat serum for 20 min at room temperature prevented non-specific binding. The sections were incubated overnight at 4 °C with antibodies iNOS (1:200), COX-2 (1:250), NF-κB p65 (1:200), IκB-α (1:00), p-IKKα/β (1:75), HO-1 (1:1000), and Nrf2 (1:100). Peroxidase (Histofine SAB-Po kit; Nichirei, Tokyo, Japan) was then used for staining.

### 4.9. Statistical Analysis

StatMate V (ATMS Co., Ltd., Tokyo, Japan) was used for statistical analyses. Parametric data were analyzed using variance (ANOVA), and an ad hoc comparison between the two treatment groups was performed using the Tukey test. Nonparametric data were analyzed using the Kruskal–Wallis test, and an ad hoc comparison between the two treatment groups was performed using the Newman–Keuls test. All data are expressed as the mean ± standard deviations. A *p*-value of less than 0.05 was considered statistically significant.

## Data Availability

The data presented in this study are available upon request from the corresponding authors.

## References

[1] Rosenbaum J.T., McDevitt H.O., Guss R.B., Egbert P.R. (1980). Endotoxin-induced uveitis in rats as a model for human disease. Nature.

[2] Chang Y.H., Horng C.T., Chen Y.H., Chen P.L., Chen C.L., Liang C.M., Chien M.W., Chen J.T. (2008). Inhibitory effects of glucosamine on endotoxin-induced uveitis in Lewis rats. Investig. Ophthalmol. Vis. Sci..

[3] Bhattacherjee P., Williams R.N., Eakins K.E. (1983). An evaluation of ocular inflammation following the injection of bacterial endotoxin into the rat foot pad. Investig. Ophthalmol. Vis. Sci..

[4] Uchida T., Honjo M., Yamagishi R., Aihara M. (2017). The anti-inflammatory effect of ripasudil (K-115), a rho kinase (ROCK) inhibitor, on endotoxin-induced uveitis in rats. Investig. Ophthalmol. Vis. Sci..

[5] Shoeb M., Zhang M., Xiao T., Syed M.F., Ansari N.H. (2018). Amelioration of endotoxin-induced inflammatory toxic response by a metal chelator in rat eyes. Investig. Ophthalmol. Vis. Sci..

[6] Yadav U.C., Ramana K.V. (2013). Endotoxin-induced uveitis in rodents. Methods Mol. Biol..

[7] de Vos A.F., van Haren M.A., Verhagen C., Hoekzema R., Kijlstra A. (1994). Kinetics of intraocular tumor necrosis factor and interleukin-6 in endotoxin-induced uveitis in the rat. Investig. Ophthalmol. Vis. Sci..

[8] Planck S.R., Huang X.N., Robertson J.E., Rosenbaum J.T. (1994). Cytokine mRNA levels in rat ocular tissues after systemic endotoxin treatment. Investig. Ophthalmol. Vis. Sci..

[9] Iwama D., Miyahara S., Tamura H., Miyamoto K., Hirose F., Yoshimura N. (2008). Lack of inducible nitric oxide synthases attenuates leukocyte-endothelial cell interactions in retinal microcirculation. Br. J. Ophthalmol..

[10] Smith J.R., Hart P.H., Williams K.A. (1998). Basic pathogenic mechanisms operating in experimental models of acute anterior uveitis. Immunol. Cell Biol..

[11] Yoshida M., Yoshimura N., Hangai M., Tanihara H., Honda Y. (1994). Interleukin-1 alpha, interleukin-1 beta, and tumor necrosis factor gene expression in endotoxin-induced uveitis. Investig. Ophthalmol. Vis. Sci..

[12] Kanai K., Itoh N., Yoshioka K., Yonezawa T., Ikadai H., Hori Y., Ito Y., Nagai N., Chikazawa S., Hoshi F. (2010). Inhibitory effects of oral disulfiram on endotoxin-induced uveitis in rats. Curr. Eye Res..

[13] Kubota S., Kurihara T., Mochimaru H., Satofuka S., Noda K., Ozawa Y., Oike Y., Ishida S., Tsubota K. (2009). Prevention of ocular inflammation in endotoxin-induced uveitis with resveratrol by inhibiting oxidative damage and nuclear factor-kappaB activation. Investig. Ophthalmol. Vis. Sci..

[14] Yadav U.C., Subramanyam S., Ramana K.V. (2009). Prevention of endotoxin-induced uveitis in rats by benfotiamine, a lipophilic analogue of vitamin B1. Investig. Ophthalmol. Vis. Sci..

[15] Sato K., Mihara Y., Kanai K., Yamashita Y., Kimura Y., Itoh N. (2016). Tyrosol ameliorates lipopolysaccharide-induced ocular inflammation in rats via inhibition of nuclear factor (NF)-κB activation. J. Vet. Med. Sci..

[16] Li Q., Verma I.M. (2002). NF-kappaB regulation in the immune system. Nat. Rev. Immunol..

[17] Silverman N., Maniatis T. (2001). NF-kappaB signaling pathways in mammalian and insect innate immunity. Genes. Dev..

[18] Surh Y.J., Chun K.S., Cha H.H., Han S.S., Keum Y.S., Park K.K., Lee S.S. (2001). Molecular mechanisms underlying chemopreventive activities of anti-inflammatory phytochemicals: Down-regulation of COX-2 and iNOS through suppression of NF-kappa B activation. Mutat. Res..

[19] Lennikov A., Kitaichi N., Noda K., Mizuuchi K., Ando R., Dong Z., Fukuhara J., Kinoshita S., Namba K., Ohno S. (2014). Amelioration of endotoxin-induced uveitis treated with the sea urchin pigment echinochrome in rats. Mol. Vis..

[20] Srivastava S.K., Ramana K.V. (2009). Focus on molecules: Nuclear factor-kappaB. Exp. Eye Res..

[21] Jin X.H., Ohgami K., Shiratori K., Suzuki Y., Hirano T., Koyama Y., Yoshida K., Ilieva I., Iseki K., Ohno S. (2006). Inhibitory effects of lutein on endotoxin-induced uveitis in Lewis rats. Investig. Ophthalmol. Vis. Sci..

[22] Park J., Kim J.T., Lee S.J., Kim J.C. (2020). The anti-inflammatory effects of angiogenin in an endotoxin induced uveitis in rats. Int. J. Mol. Sci..

[23] Maines M.D. (1997). The heme oxygenase system: A regulator of second messenger gases. Annu. Rev. Pharmacol. Toxicol..

[24] Suematsu M., Wakabayashi Y., Ishimura Y. (1996). Gaseous monoxides: A new class of microvascular regulator in the liver. Cardiovasc. Res..

[25] Ohta K., Kikuchi T., Arai S., Yoshida N., Sato A., Yoshimura N. (2003). Protective role of heme oxygenase-1 against endotoxin-induced uveitis in rats. Exp. Eye Res..

[26] Kapitulnik J., Maines M.D. (2009). Pleiotropic functions of biliverdin reductase: Cellular signaling and generation of cytoprotective and cytotoxic bilirubin. Trends Pharmacol. Sci..

[27] Otterbein L.E., Bach F.H., Alam J., Soares M., Tao Lu H., Wysk M., Davis R.J., Flavell R.A., Choi A.M. (2000). Carbon monoxide has anti-inflammatory effects involving the mitogen-activated protein kinase pathway. Nat. Med..

[28] Chen L.G., Zhang Y.Q., Wu Z.Z., Hsieh C.W., Chu C.S., Wung B.S. (2018). Peanut arachidin-1 enhances Nrf2-mediated protective mechanisms against TNF-α-induced ICAM-1 expression and NF-κB activation in endothelial cells. Int. J. Mol. Med..

[29] Alam J., Stewart D., Touchard C., Boinapally S., Choi A.M., Cook J.L. (1999). Nrf2, a Cap’n’Collar transcription factor, regulates induction of the heme oxygenase-1 gene. J. Biol. Chem..

[30] Kobayashi A., Kang M.I., Watai Y., Tong K.I., Shibata T., Uchida K., Yamamoto M. (2006). Oxidative and electrophilic stresses activate Nrf2 through inhibition of ubiquitination activity of Keap1. Mol. Cell. Biol..

[31] Fujino M., Nishio Y., Ito H., Tanaka T., Li X.K. (2016). 5-aminolevulinic acid regulates the inflammatory response and alloimmune reaction. Int. Immunopharmacol..

[32] Ishizuka M., Abe F., Sano Y., Takahashi K., Inoue K., Nakajima M., Kohda T., Komatsu N., Ogura S.I., Tanaka T. (2011). Novel development of 5-aminolevurinic acid (ALA) in cancer diagnoses and therapy. Int. Immunopharmacol..

[33] Rodriguez B.L., Curb J.D., Davis J., Shintani T., Perez M.H., Apau-Ludlum N., Johnson C., Harrigan R.C. (2012). Use of the dietary supplement 5-aminiolevulinic acid (5-ALA) and its relationship with glucose levels and hemoglobin A1C among individuals with prediabetes. Clin. Transl. Sci..

[34] Miura M., Ito K., Hayashi M., Nakajima M., Tanaka T., Ogura S. (2015). The effect of 5-aminolevulinic acid on cytochrome P450-mediated prodrug activation. PLoS ONE.

[35] Liu C., Zhu P., Fujino M., Zhu S., Ito H., Takahashi K., Nakajima M., Tanaka T., Zhuang J., Li X.K. (2019). 5-ALA/SFC Attenuated binge alcohol-induced gut leakiness and inflammatory liver disease in HIV transgenic rats. Alcohol. Clin. Exp. Res..

[36] Hara T., Koda A., Nozawa N., Ota U., Kondo H., Nakagawa H., Kamiya A., Miyashita K., Itoh H., Nakajima M. (2016). Combination of 5-aminolevulinic acid and ferrous ion reduces plasma glucose and hemoglobin A1c levels in Zucker diabetic fatty rats. FEBS Open Bio.

[37] Shimura M., Nozawa N., Ogawa-Tominaga M., Fushimi T., Tajika M., Ichimoto K., Matsunaga A., Tsuruoka T., Kishita Y., Ishii T. (2019). Effects of 5-aminolevulinic acid and sodium ferrous citrate on fibroblasts from individuals with mitochondrial diseases. Sci. Rep..

[38] Ito H., Nishio Y., Hara T., Sugihara H., Tanaka T., Li X.K. (2018). Oral administration of 5-aminolevulinic acid induces heme oxygenase-1 expression in peripheral blood mononuclear cells of healthy human subjects in combination with ferrous iron. Eur. J. Pharmacol..

[39] Liu C., Wang Z., Hu X., Ito H., Takahashi K., Nakajima M., Tanaka T., Zhu P., Li X.K. (2021). 5-aminolevulinic acid combined with sodium ferrous citrate ameliorated lupus nephritis in a mouse chronic graft-versus-host disease model. Int. Immunopharmacol..

[40] Otaka Y., Kanai K., Okada D., Nagai N., Yamashita Y., Ichikawa Y., Tajima K. (2023). Effects of oral 5-aminolevulinic acid on lipopolysaccharide-induced ocular inflammation in rats. Vet. Sci..

[41] Ohgami K., Ilieva I., Shiratori K., Koyama Y., Jin X.H., Yoshida K., Kase S., Kitaichi N., Suzuki Y., Tanaka T. (2005). Anti-inflammatory effects of aronia extract on rat endotoxin-induced uveitis. Investig. Ophthalmol. Vis. Sci..

[42] Hou J., Zhang Q., Fujino M., Cai S., Ito H., Takahashi K., Abe F., Nakajima M., Tanaka T., Xu J. (2015). 5-aminolevulinic acid with ferrous iron induces permanent cardiac allograft acceptance in mice via induction of regulatory cells. J. Heart Lung Transplant..

[43] Xu Y., Chen W., Lu H., Hu X., Li S., Wang J., Zhao L. (2010). The expression of cytokines in the aqueous humor and serum during endotoxin-induced uveitis in C3H/HeN mice. Mol. Vis..

[44] Tilton R.G., Chang K., Corbett J.A., Misko T.P., Currie M.G., Bora N.S., Kaplan H.J., Williamson J.R. (1994). Endotoxin-induced uveitis in the rat is attenuated by inhibition of nitric oxide production. Investig. Ophthalmol. Vis. Sci..

[45] Mandai M., Yoshimura N., Yoshida M., Iwaki M., Honda Y. (1994). The role of nitric oxide synthase in endotoxin-induced uveitis: Effects of NG-nitro L-arginine. Investig. Ophthalmol. Vis. Sci..

[46] Bellot J.L., Palmero M., García-Cabanes C., Espí R., Hariton C., Orts A. (1996). Additive effect of nitric oxide and prostaglandin-E2 synthesis inhibitors in endotoxin-induced uveitis in the rabbit. Inflamm. Res..

[47] Jin X.H., Ohgami K., Shiratori K., Suzuki Y., Koyama Y., Yoshida K., Ilieva I., Tanaka T., Onoe K., Ohno S. (2006). Effects of blue honeysuckle (*Lonicera caerulea* L.) extract on lipopolysaccharide-induced inflammation in vitro and in vivo. Exp. Eye Res..

[48] Kanai K., Ito Y., Nagai N., Itoh N., Hori Y., Chikazawa S., Hoshi F., Higuchi S. (2012). Effects of instillation of eyedrops containing disulfiram and hydroxypropyl-β-cyclodextrin inclusion complex on endotoxin-induced uveitis in rats. Curr. Eye Res..

[49] Kalariya N.M., Shoeb M., Ansari N.H., Srivastava S.K., Ramana K.V. (2012). Antidiabetic drug metformin suppresses endotoxin-induced uveitis in rats. Investig. Ophthalmol. Vis. Sci..

[50] Shi X., Zhu S., Jin H., Fang J., Xing X., Wang Y., Wang H., Wang C., Niu T., Liu K. (2021). The anti-inflammatory effect of KS23, a novel peptide derived from globular adiponectin, on endotoxin-induced uveitis in rats. Front. Pharmacol..

[51] Yang X., Jin H., Liu K., Gu Q., Xu X. (2011). A novel peptide derived from human pancreatitis-associated protein inhibits inflammation in vivo and in vitro and blocks NF-kappa B signaling pathway. PLoS ONE.

[52] Ihaka R., Gentleman R. (1996). R: A Language for Data Analysis and Graphics. J. Comput. Graph. Stat..

[53] Behar-Cohen F.F., Savoldelli M., Parel J.M., Goureau O., Thillaye-Goldenberg B., Courtois Y., Pouliquen Y., de Kozak Y. (1998). Reduction of corneal edema in endotoxin-induced uveitis after application of L-NAME as nitric oxide synthase inhibitor in rats by iontophoresis. Investig. Ophthalmol. Vis. Sci..

